# Vanishing Girls, Mysterious Blacks

**DOI:** 10.1177/2041669518786740

**Published:** 2018-07-20

**Authors:** Jan Koenderink, Andrea van Doorn, Johan Wagemans

**Affiliations:** Laboratory of Experimental Psychology, University of Leuven (KU Leuven), Belgium; Experimental Psychology, Helmholtz Institute, Utrecht University, the Netherlands; Experimental Psychology, Helmholtz Institute, Utrecht University, the Netherlands; Laboratory of Experimental Psychology, University of Leuven (KU Leuven), Belgium

**Keywords:** amodal contour, lost edges, passages, macchia

## Abstract

Participants had to indicate the location of points on what might be called “amodal contours” in some works of art. The works represented mutually quite different cases. In one case, there were not even scattered modal cues, thus the amodal contour had to be hallucinated on the basis of generic familiarity. Here, observers indicated coherent geometrical structures (to a good approximation a smooth curve), although at idiosyncratic locations. In another case, we presented an ambiguous image that led to much more “fuzzy” amodal completions. We also presented an image that had at least some similarity to a configuration treated by Kanizsa. Here, observers were coherent and they mutually agreed, so the scarce modal cues apparently largely dictated the awareness.

## Introduction

We consider the topic of “amodal completion” (Briscoe, 2011; De Wit & van Lier, 2002; Nanay, 2010; Sekuler & Murray, 2001; van Lier & Gerbino, 2015) from the perspective of the visual arts and from the background of visual perception. The latter background is considered common knowledge to the readers of this journal. We provide a summary introduction to the topic from the perspective of the visual arts.

Note that we use the terms modal/amodal in a perhaps slightly unusual, generic sense. That is to say, we use amodal for any spatial element (such as a line or a contour) in visual awareness that is not immediately specified by a corresponding spatial element in the stimulus. We prefer such a definition because it is objective. Thus, an amodal line is a perceived line (a *quale*), which is not immediately specified by some contrasting region, that is elongated in one direction, rather narrow in an orthogonal direction in the stimulus (*a property of the image*). Likewise, an amodal contour is a perceived contour (a *quale*) for which there does not exist a relatively sharp, relatively straight transition between two more or less uniform, extended, and contrasting regions in the stimulus (*a property of the image*). Apparently, “illusory” might be used interchangeably with “amodal.” However, we avoid this term because of unwanted connotations (Koenderink, 2017). In case an amodal contour is seen as occluding, the occluded part would also be amodal, but in an ontologically different sense. This plays no role in this experiment. For further terminological discussion, see van Lier and Gerbino (2015); Scherzer and Ekroll (2015); and Kanizsa (1955) on “virtual lines.”

### Passages and Lost Contour

**Figure 1.**
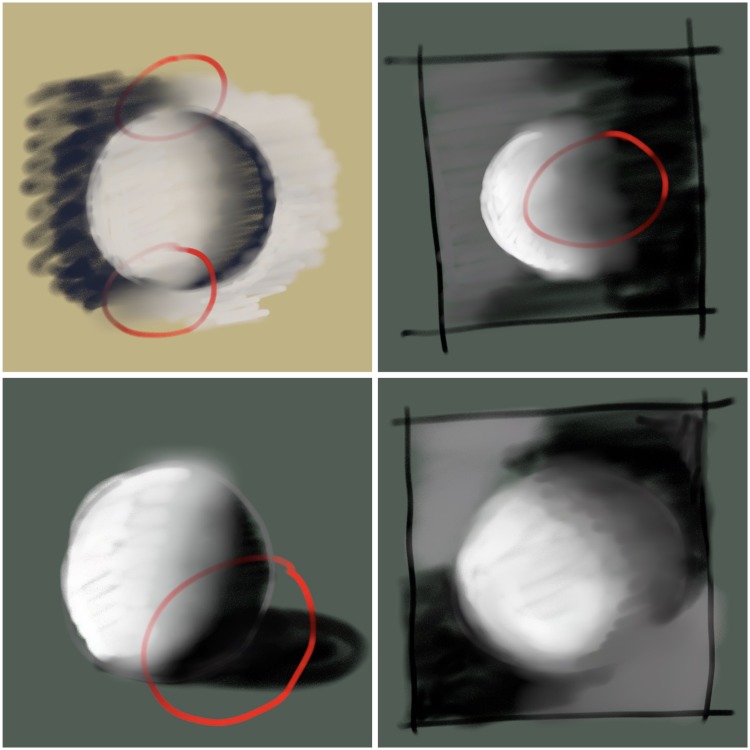
Some initial designs in pastels. Top left: contours lost and found through counter-shaded background. Top right: passage created through mysterious dark. Bottom left: passage created through merge of body and cast shadow. Bottom right: object held in gripping darks allows for creation of possibly useful passages.

In planning an image, the artist typically starts with a rough design to settle the composition. This would typically look like a simple abstract sketch (Figure 1). One thinks in terms of two or three tones (darks, lights, and perhaps mid-tones), disregarding the scarce pure whites and blacks. The latter would be added in a final state as a kind of spice, they are treated as accents. In contradistinction, the two or three major tones cover large areas. These areas should be of simple shape, preferably connected and together they make up the major composition. One area will dominate the final picture, its shape is the macchia^1^ (or tâche) (Boime, 1993; Broude, 1987; Imbriano, 1868). The macchia is usually light and located at a central (though slightly off) location since that “attracts the eye.” At that stage, one considers such things as dominating shapes, body shadows (mezzo-macchia, the “bed bug line”^2^ or night-day terminator), and cast shadows (Cateura, 1995; Clifton, 1973; Guptill, 1928; Jacobs, 1986). They may be roughly sketched in. Perhaps surprisingly, edges may both unite abutting regions and serve to split them (Brentano’s plerosis; Agostini & Galmonte, 2000; Brentano, 1874; Koenderink, van Doorn, Pinna, & Wagemans, 2016).

On this skeleton structure, one starts looking for possible “passages” that are bridges between mutually disjunct macchie of (usually) darks or (possibly) lights. One plans a scheme of edge qualities, including the “lost and found” edges (Cateura, 1995; Clifton, 1973; Jacobs, 1986). Passages are a planned and important part of painting. Here, we meet with the amodal contours.

A passage is a lost edge. The edge is usually lost in a mysterious dark. An example would be the unlit side of a face in a portrait that smoothly blends into a dark background or the unlit side of an object that merges with the object’s cast shadow.

Lost and found contours often occur (or rather: are sought for, because nothing is a mere random coincidence in a painting, all is planned) in the case of roughly convex, volumetric bodies in front of some background (Koenderink et al., 2016). The body will have a lit and an unlit side (straddling the bed bug line), whereas the tonal gradients in the background are largely to the artist’s disposal. Some gradient will be planned that runs counter to the shading of the object, leading to places where the tone of the shading matches that of the background, thus causing the edge to be lost. The edge is soon “found” again, albeit in opposite polarity. Of course, the background has other functions, such as “holding” the dominant object in gripping darks, yielding an attractive pattern, and so forth. That is why a planning stage is a necessity.

The artist assumes full control over the background, designing it so as to create the planned passages and lost contours. Gripping or holding darks or lights tend to be *designed*, rather than *found* in the scene (if drawn from nature).

These are standard devices that are likely to play a role in virtually any painting. But, of course, there are many other ways to lose contours. The artist will always look for them or create possibilities for them. The reason is simple enough. Passages and lost contours engage the creative vision of the observer of the work, thus they create interest and help fight boredom. Moreover, anything the spectator contributes (Gombrich’s (1950) “beholder’s share”) will be a major contribution to the work. There is no way the painter could beat that in paint. Passages and lost contours add depth and atmosphere by setting the work sharply apart from silhouettes, or the outlined areas in children’s coloring books in which passages and lost contours are avoided and as a consequence look flat and boring.

### The Experiment

**Figure 2.**
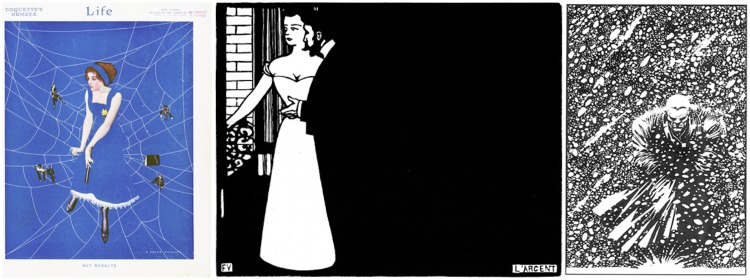
Full versions of the art works used as stimuli. In the experiment, cutouts were used in order to maximize the areas depending on their artistic effect (*passages*) on amodal contours. From left to right, Coles Phillips (1880–1927), frontispiece of Life Magazine (“Coquette’s Number” Vol. LVIII, No. 1504, August 24, 1914), Félix Valloton (1865–1925), “L’Argent” (woodcut 1897, from Intimités), and Frank Miller (born 1957), frame from his comics book “Sin City” (“The Hard Goodbye,” Dark Horse Comics, Milwaukie, OR, 1991–1992).

In this experiment, we use three works that show rather extreme examples of lost contours that are arrived at through very different techniques (Figure 2).

The first example uses occlusion of lines (a relatively rare technique, tricky to use), the second example uses a standard (though quite extreme) method of contours lost in mysterious darks, and the third example uses a (rather unique) method of losing a contour in a random texture. These by no means exhaust common techniques, and it would not be that hard to find different examples for each of the three.

In the experiment, we try to find the lost contour as contributed by the awareness of a number of observers. Of course, there is no such a thing as ground truth, the contour is “seen,” but it is not physically present in the work (Perky, 1910). We consider the following two major questions:

1. Can observers indeed hallucinate the missing contour at all? Are they coherent in their experience over time?
2. Do observers mutually agree in these experiences? That is to say, does the artist succeed in letting various observers hallucinate the same, or at least similar, nonexisting contours?

## Methods

### Stimuli

The stimuli were images of well-known artworks (Figure 2), downloaded from the Internet. We will indicate them by way of the artist’s names, that is Coles Phillips (or just Phillips), an American illustrator (Schau, 1975; Tse, 2017), famous for his “vanishing girls” (due to use of amodal contours); Félix Valloton (or just Valloton), Swiss painter and graphics artist (Ducrey, 1989), working mostly in Paris, well known for his use of mysterious blacks and passages in woodcuts; and Frank Miller (or just Miller), an American comics artist (Miller, 2018), famous from the movie *Sin City*, well known for his use of lost contour in high-contrast pen drawings.

All are prints, the Valloton possibly printed by the artist (a woodcut), the other two by the printing press for the masses. Phillips probably used watercolor media^3^ (Quiller, 1986) and inks, Miller India Ink, the techniques are not important here. What is relevant is that all works use only a few colors over large areas or in thin lines. There are no modulations as one would find in oil painting, for instance.

The images were presented on a computer display on a dark background and were viewed from a normal viewing distance of ca. 40 cm. We used a MacBook Pro 15″ and performed the experiment in a darkened room.

### Observers

Observers were 22 (in their 20 s, about matched for gender) students (bachelor, master, and PhD) and postdocs from the University of Leuven (KU Leuven). They had no specific connections with the visual arts, but were familiar with the conventional “visual effects” as demonstrated in courses on perception.

## Experiment

### Design

The stimuli were selected because of the presence of roughly vertical, isolated, rather long amodal contours. This makes it possible to search for the location of the amodal contour along a horizontal line. The task of the participants was to indicate this location (Guttman & Kellman, 2004). We draw a thin horizontal line (orange) over the full width of the stimulus and put a small, but easily visible dot on it. The participant can use the mouse (using only its horizontal position, there is no need to hit the dot) to move the dot and place it on the amodal contour. If the position is judged satisfactory, the participant hits the ENTER key, which concludes the setting. During the adjustment phase, the participant may press the SPACE bar to remove the horizontal line, the dot always remaining visible. Participants are asked to do at least a hundred settings per stimulus and at most spend an hour on the total task.

**Figure 3.**
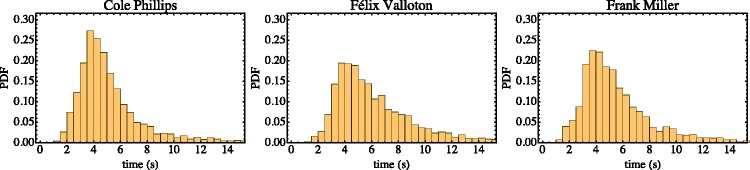
Distribution of response times for the three stimuli. Observers take 5.5 seconds (median, interquartile range: 4.3–7.9) per setting for the Phillips stimulus, 4.6 seconds (IQR: 3.6–6.1) for the Valloton stimulus, and 5.0 seconds (IQR: 3.8–6.8) for the Miller stimulus.

We find that observers take about 5 seconds per setting (Figure 3). There is no real difference in response times for the three stimuli, although the tasks appear rather different from each other in kind.

It was decided to perform measurements at random heights and to alternate between stimuli (in fixed order) at the conclusion of each setting. This has obvious advantages and disadvantages. It also influences the possible ways of analysis. For instance, any given height is unlikely to reappear and participants do not visit the same heights.

**Figure 4.**
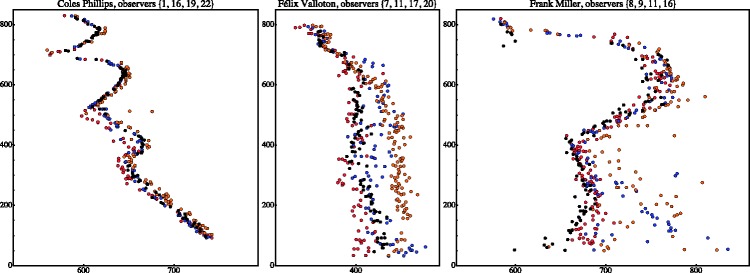
Raw results for the case of some selected observers. The horizontal scale has been magnified by a factor of three for easy comparison. This yields a generic view of the results, all other observers are somewhere in between these cases.

Selected raw results are presented in Figure 4. (Notice that the horizontal scale in the scatterplot has been expanded by a factor of three!)

A few aspects are evident at first blush:

*First*, if there is a “real” contour, all participants agree very precisely. Good examples are the arm of the girl in the Phillips stimulus, the white collar of the man in the Valloton stimulus, and the arm of the person in the Miller stimulus.
*Second*, if there “is no clue” as to where the lost contour might be, settings clearly diverge very significantly. A good example is the Valloton stimulus, where the region that contains the amodal contour is just a uniform, solid black (an extreme example of a passage). Interestingly, individual participants produce well-defined contours, it is the location of these contours that differs.
*Third*, if there is pictorial ambiguity, as in the Miller stimulus, where the texture due to the “snow” and the person’s clothes intermingle, one spots both interobserver differences in location and intraobserver scatter.

A formal analysis is provided here.

### Analysis

There are many alternative ways to analyze the results formally. For the presentation used here, we use only the simplest and most direct.

**Figure 5.**
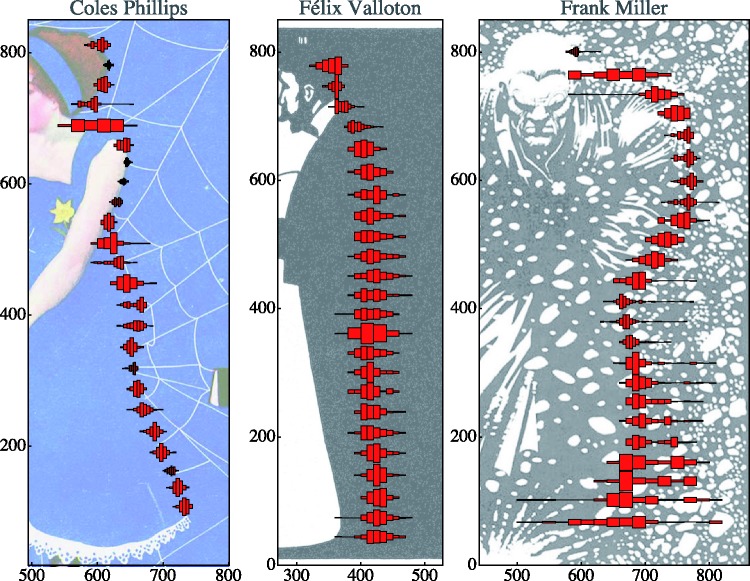
Histograms in slices of height containing 100 settings each, pooled over all observers. (Optimal bin widths are automatically assigned by way of one-level recursive approximate Wand binning.) This yields a concise overview of the structure of the data, especially the qualitative differences for the three cases are clearly exhibited.

For an initial analysis,^4^ we divide the height of the contour into 24 slices, such that each slice contains a hundred settings. We consider the histograms of the settings (Figure 5).

**Figure 6.**
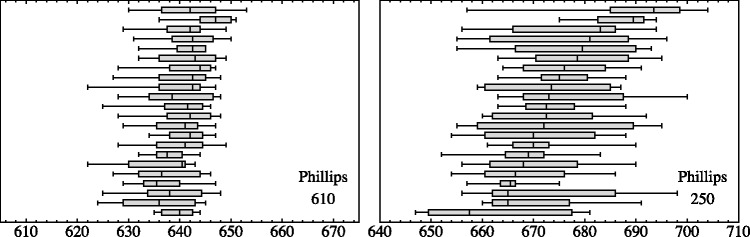
Full range and quartiles for all observers, for two selected heights (610 and 250 pixels, see Figure 5) and the Phillips stimulus. Observers have been sorted by the median in the plot at right. Both plots have the same horizontal scale. The left height is at the arm, the right height at the middle part of the skirt.

Consider the Phillips stimulus (Figure 6). In the slices representing results for the arm, the standard deviations are small, just a few pixels and participants agree very well. Evidently, the contour is very well determined, because in this case, it is physically represented in the image. It is of more immediate interest to consider the slices that represent the amodal contour. Here, the standard deviations per observer are somewhat larger, of the order of 10 pixels. The results for the various observers agree quite well, typically within a fraction of the standard deviation per observer, for two of the slices just a little over that standard deviation. Thus, the amodal contour is also well determined and very similar for all observers.

**Figure 7.**
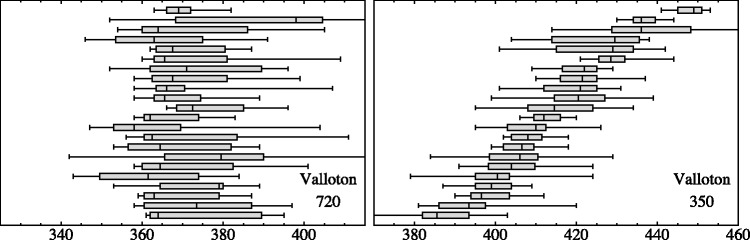
Full range and quartiles for all observers, for two selected heights (720 and 350 pixels, see Figure 5) and the Valloton stimulus. Observers have been sorted by the median in the plot at right. Both plots have the same horizontal scale. The left height is at the white collar, the right height at about halfway the height of the figure.

For the Valloton stimulus (Figure 7), the case is different. Here, the actual contour is only physically indicated at one point, the white collar of the man. Indeed, all observers agree quite well at that point. For most of the height range, there is no indication at all though. Here, observers diverge quite a bit. It is interesting that they are individually quite coherent, whereas they mainly differ among each other. This is somewhat understandable in view of our own impression that the man has a sharp contour at the back side, although that contour is apparently lost in the mysterious black. The participants are likely to have similar impressions, their contours being quite well defined, although mutually rather distinct.

**Figure 8.**
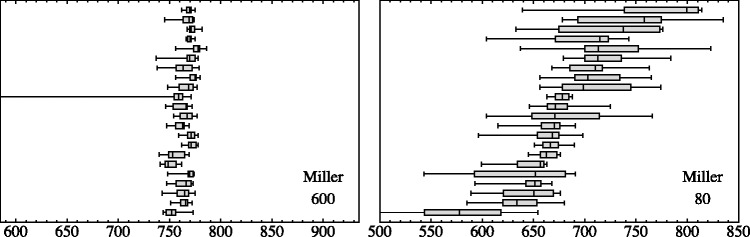
Full range and quartiles for all observers, for two selected heights (600 and 80 pixels, see Figure 5) and the Miller stimulus. Observers have been sorted by the median in the plot at right. Both plots have the same horizontal scale. The left height is at the arm, the right height very low in the image. (In the left plot, one spots the effect of an outlier due to the fact that an observer continued to the next trial without having completed the present one: We did not remove any of the (very few) outliers in this study.)

The Miller case is different again (Figure 8). Here, the arm has a fairly well-defined contour due to obvious pictorial cues, whereas the lower part of the legs region hardly offers much of a cue at all. This is worse than in the Valloton case because there is a mysterious *texture* instead of a mysterious *black*. It is obvious from the result that the participants indeed attempt to use that texture, but that they interpret it in different ways.

**Figure 9.**
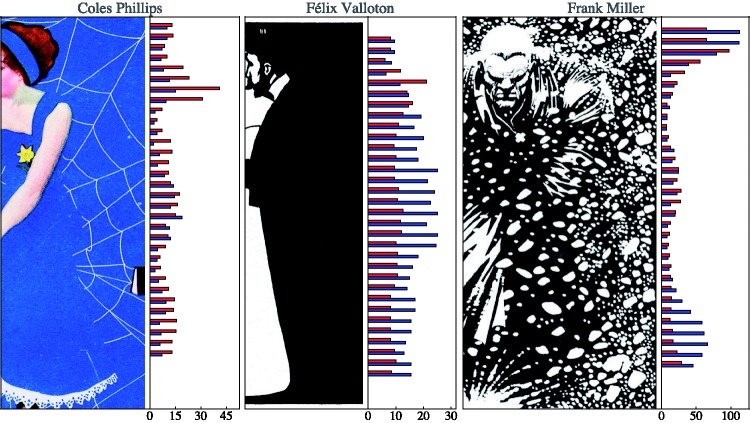
An analysis of the spread as a function of height. The red bars are the median of the interquartile ranges over all participants, and the blue bars are the interquartile range of the medians of the locations over all participants. The length of the bars is given in terms of pixels (the height of the stimuli is 850 pixels). Notice that the scales are very different for the three cases!

A more quantitative analysis involves both the location of the contour and the spread. Here, we sample at 32 height levels (Figure 9). At each height, we select the 10 nearest (by height, remember the heights are uniformly distributed random values) responses for each participant. We compute the median and the interquartile range (IQR) of the horizontal locations for each observer.

The IQR of the set of individual medians for each participant is a measure of the interobserver variability. It is plotted as the blue bars in Figure 9. The median of the set of individual IQRs for each participant is a measure of the intraobserver variability. It is plotted as the red bars in Figure 9. The length of the red bars is a measure of individual variability. The length of the blue bars is a measure of the variability over participants.

When the red bars are equal or larger than the blue bars, the responses of the participants mutually agree. When the blue bars are larger than the red bars, this signifies that the participants widely disagree among each other.

Notice that the *scales* are very different for the data plotted in Figure 9:

*For the Phillips case*, the maximum of the medians of the IQRs (red bars) is 41 pixels, whereas the maximum of the IQRs of the medians (blue bars) is 19 pixels.
*For the Valloton case*, the maximum of the medians of the IQRs (red bars) is 21 pixels, whereas the maximum of the IQRs of the medians (blue bars) is 25 pixels.
*For the Miller case*, the maximum of the medians of the IQRs (red bars) is 97.5 pixels, whereas the maximum of the IQRs of the medians (blue bars) is 112.5 pixels.

These plots (Figure 9) yield an excellent overview of the results, especially when combined with the examples of individual data presented in Figure 4.

For the Phillips stimulus, the locations per observer are well defined; moreover, the spread of locations over observers is even less than the spread per participant. Apparently, all observers consider this amodal contour somehow well defined and they mutually agree on its location. This neatly fits expectations from vision science (Kanizsa, 1955; Soriano, Spillmann, & Bach, 1996).

In contradistinction, for the Valloton stimulus the locations are idiosyncratic, the spread over observers being larger than the spread per individual participant. Apparently, all observers consider this amodal contour reasonably well defined, but they do not mutually agree on exactly where it is. Here, we deal essentially with a hallucination, no doubt based on generic familiarity^5^ (Hatton, 1904; Lohmar, 2014).

For the Miller stimulus, we see a mixed case. In the range of the upper arm, it resembles the Phillips case, in the range of the legs, the Valloton case. In the latter range, observers are individually very uncertain and they also differ a lot among each other (compare Figure 4, right).

## Conclusions

We determined amodal contours for several observers in three art works. The works are mutually quite different, and we indeed find striking differences in responses. In the visual arts, creators of drawings, paintings, … , count on the “beholder’s share” (Gombrich, 1950) and intentionally use passages, lost edges, and lost-and-found contours, counting on the viewer to provide amodal elements (Kandinsky, 1926). Such amodal elements often differ in kind from the amodal elements routinely encountered in vision science (Craik, 1966; Kanizsa, 1955; Soriano, Spillmann, & Bach, 1996). In this experiment, only one instance (the Phillips image) is somewhat close to a familiar case regarded in vision science. Another case (the Valloton image) requires what might perhaps more properly be called *hallucination* than *amodal completion* based on scattered visual cues—there are none of the latter. Yet it is the case perhaps most commonly encountered in the visual arts, a passage created through a mysterious black.

Of course, the images taken from the arts differ from the standard vision science examples in that they have strong semantic content. Such content is not entirely lacking in the latter case though, there the visual objects tend to be familiar geometrical objects such as straight lines (e.g., Kanizsa’s abutting gratings) or regular polygons (e.g., Kanizsa’s triangle). In both cases, familiarity is also quite likely to play a role.

From the perspective of vision science, it is perhaps interesting to take note of the fact that the visual arts use amodal elements intentionally and profusely and that the range of qualitatively different types most likely exceeds that of the types considered by vision scientists. Of course, such amodal elements in the arts are less conspicuous than those in science. Whereas the latter are often presented as demos, the former tend to be artfully hidden. They are part of the artist’s toolbox.

From the perspective of the arts, it is of interest to note that amodal elements may well lead to very different visions in spectators. This is not necessarily a bad thing and indeed is more often considered to enrich a work. However, it is no doubt of interest to be aware of the spectrum of possible visual readings.

## Notes

1 Macchia (plural macchie) literally means “blotch,” “patch,” “spot.” For the macchiaioli, the term had various relevant connotations, one of these was the implication of a sketchy, or unfinished look—which was actually sought for. Another was a reference to plein-air painting (in many respects, the macchiaioli were protoimpressionists—roughly a decade ahead of Monet; Boime, 1993; Broude, 1987; Imbriano, 1868), *macchia mediterranea* standing for a kind of scrubland, thus “*darsi alla macchia*” meaning something like “hiding in the bushes.” Here, we intend only the use of blocky color.

2 On this technical term, in common use in the visual arts, see https://www.sightsize.com/articles/beware-of-the-bed-bug-line/ and https://gurneyjourney.blogspot.com/2010/02/light-and-form-part-1_15.html.

3 A reviewer read this as “watercolor painting” (or aquarelle) implying paints without white filler, using the color of the paper for “white.” Here, we intend any medium using some glue in water as solvent, instead of oils or acrylic medium. This includes gouache, casein, and so forth, in which cases white pigment is mixed in the binder to increase opacity. In this case, we would guess that Phillips used a kind of gouache, which is ideal and often used for illustration purposes.

4 In this analysis, we largely ignore the parts in which the contour was not approximately vertical. This mainly occurs in shoulder areas. Taking obliquity into account would unnecessarily complicate the discussion.

5 This includes familiarity with the draped human figure, of course. However, there are also a variety of generic structures of an abstract nature. For instance, elongated objects are bounded by (among more) two “parallel” contours, one on each side. These contours are never *geometrically* parallel, but they certainly correspond in some way, a way that tends to suggest properties of the volume bounded by them. There is quite a bit of lore in the arts on how to treat such contour pairs. They should “echo” each other in various exciting ways (avoiding “garden hose” or “sausage” looks) and are necessarily treated as a pair. To treat them as separate items immediately shows up the artist as of lesser understanding. If one of a pair is missing, it still echoes the visible one.
